# Medical comorbidity and projected survival in patients admitted to a specialist addictions in-patient unit

**DOI:** 10.1192/pb.bp.115.051987

**Published:** 2016-10

**Authors:** Daniel V. Mogford, Rebecca J. Lawrence

**Affiliations:** 1 NHS Lothian, Edinburgh, UK

## Abstract

**Aims and method** To investigate the burden of medical comorbidity in a population receiving in-patient treatment for drug and alcohol problems. All patients admitted over a 6-month period were included in the data-set. We recorded diagnostic information on admission that allowed the calculation of predicted 10-year survival using a previously validated comorbidity index.

**Results** Despite the majority of the sample having a predicted 10-year survival chance of greater than 75%, a sizeable minority (16.7%) are carrying a high burden of medical comorbidity, with a predicted 10-year survival chance of less than 50%. More than half (55.2%) of these patients were under the age of 55. Chronic respiratory disease was the most frequent diagnosis.

**Clinical implications** In-patient substance misuse units serve a complicated group of patients, whose needs are met by active medical input, resident medical cover and effective liaison with general hospitals. This is important when planning and commissioning treatment services. The high burden of respiratory disease suggests the utility of robust smoking cessation interventions among this population.

It is well established that there is a wide range of social, psychological and physical harms associated with chronic substance misuse.^1^ It is also clear that, for people with mental illness, substance misuse is an independent risk factor for physical illness.^2^ Specialist drug and alcohol treatment providers located within psychiatric services are uniquely placed to deal with this complex population, and require the resources and skills to effectively diagnose, monitor and offer interventions for significant physical frailty. In an environment where healthcare is increasingly being provided in a community-based setting, it is important that the utility of an in-patient unit is well defined.

This study aims to characterise the burden of medical comorbidity in patients admitted to an in-patient drug and alcohol treatment service within a large psychiatric hospital. These population data are invaluable to the process of planning and commissioning services. We also hope that the data will help to identify targets for intervention beyond what is offered for the primary problem of substance misuse.

We are not aware of other attempts to measure the burden of comorbid medical illness in a heterogeneous population of patients with substance misuse.

## Method

We conducted the present study as a prospective survey. We did not seek to demonstrate the efficacy of interventions, but rather to explore the population's characteristics.

Measurement of comorbidity is broadly divided into simple counts of disease or prescribed medications, and validated measures that apply weighting to a specific subset of conditions based on their contribution to morbidity, mortality or resource utilisation. A comorbidity index serves to distil significant complexity down to a single score to allow comparison with other patients.^3^

We used the Charlson Comorbidity Index (CCI), a cumulative disease burden index, as it has the advantage of being short and easily applied based on the information already obtained as part of a standard medical admission. The CCI was originally developed using a cohort of 559 medical patients, and was intended to estimate the likelihood that a clinical population would survive to benefit from a given intervention by providing a relative risk of dying.^4^ Its validity as a combined age–comorbidity index was studied using an additional cohort of 226 surgical patients.^5^ The index records 16 diagnoses that have predictive value when calculating projected survival. The central role that age plays in predicted survival is reflected in the addition of an age-based score to the raw CCI.

We used an extended version of the CCI that includes four additional items that predict healthcare cost, but are excluded from the calculation of predicted 10-year survival. It was further validated using health data for 5861 patients.^6^ We chose to use this version for the additional breadth of common conditions captured, namely hypertension, skin ulcers or recurrent cellulitis, treatment with warfarin and the presence of depression.

The CCI has been used across a broad range of clinical settings and populations, and is considered valid and reliable for estimating the burden of medical comorbidity in clinical research.^7^ It has been shown to predict use of in-patient and out-patient services, length of admission to hospital and hospital care costs.^8^ Its ultimate output is a predicted probability of survival at 10 years from data collection.

Ethics approval was not required. Approval to carry out the study was granted by the local quality improvement team.

### Participants

Participants included all those admitted over a 6-month period (26 May to 27 November 2014) to a specialist in-patient assessment and detoxification unit in a large psychiatric hospital in a major Scottish city. The unit provides assessment, medical detoxification and stabilisation for patients dependent on alcohol, opioids and, less commonly, other substances including benzodiazepines, stimulants and novel psychoactive substances (NPS). Patients who are admitted are unsuitable for community-based treatment owing to a combination of medical, psychiatric or social complexities. Referrals to the unit come from community substance misuse teams operating within the local National Health Service (NHS) health board, a catchment population of over 800 000 covering Edinburgh and the Lothians, the second-largest residential population in Scotland.

The population referred for in-patient management of substance misuse is a subset of the much larger population routinely treated in community-based services. As a group, it presents particular challenges, whether it be due to the degree of dependence, dependence on multiple substances, lack of social supports, chaotic social circumstances, history of physical complications occurring during treatment or physical frailty. The sample we present is not therefore representative of the full range of people seeking treatment within substance misuse services.

In total, data for 175 admissions were collected.

### Data collection

All patients admitted for in-patient management receive a medical clerking as part of routine practice. This includes a full psychiatric history, substance misuse history, medical history, physical examination and appropriate investigations. A subset of these routinely collected data was used in the present study.

A brief, paper recording tool was created for inclusion in the admission. The full range of usual data sources were used to complete the tool (patient report, electronic patient record, previous discharge and out-patient letters, and general-practitioner clinical summaries). Each item of the CCI included a brief explanation with diagnosis boundaries where appropriate, and a tick box was used to indicate the presence of the condition. In addition to the CCI items, the reason for admission, substance misuse diagnoses, any other psychiatric diagnoses, medication count and presence or absence of chronic pain were also recorded. These additional items provided basic background data that were not easy to obtain from the patient electronic recording system. It was important that the data collection process did not add to the workload of admitting doctors, nor change existing clinical practice.

Diagnoses, whether medical or psychiatric, were not independently validated using diagnostic tests or instruments, but were discussed on a daily basis at the ward meeting with the consultant psychiatrist and the multidisciplinary team.

## Results

In total, 175 patients were admitted during the 6-month period of data collection. The demographic profile, purpose of admission, substance misuse diagnosis and the presence of a comorbid psychiatric diagnosis or chronic pain are summarised in Table 1.

**Table 1:** Study group characteristics

Age, mean (range)	44	(19–73)

Male, *n* (%)	111	(63.4)

Female, *n* (%)	64	(36.6)

Purpose of admission, *n* (%)		
Alcohol detoxification	139	(79.4)
Buprenorphine conversion	18	(10.3)
Opiate detoxification	9	(4)
Benzodiazepine detoxification	3	(1.7)
Cognitive assessment	2	(1.1)
Respite	2	(1.1)
NPS detoxification	2	(1.1)

Substance misuse diagnosis (ICD-10), *n* (%)		
Alcohol dependence (F10.2)	151	(86.3)
Opioid dependence (F11.2)	46	(26.3)
Benzodiazepine dependence (F13.2)	31	(17.7)
NPS harmful use (F19.1)	6	(3.4)
Kratom^a^ harmful use (F19.1)	1	(1.1)
Cannabis harmful use (F12.1)	1	(1.1)

Comorbid psychiatric diagnosis, *n* (%)	107	(61.1)

Comorbid chronic pain, *n* (%)	37	(21.1)

Cigarette smoking, *n* (%)	138	(78.9)

NPS, novel psychoactive substances.

a. Kratom is a tree native to Southeast Asia and is consumed for its μ-opioid receptor agonist properties

The most common reason for admission was alcohol detoxification, either as a standalone intervention or, for a small number of patients (*n* = 5, 2.9%), in combination with another intervention, such as cognitive assessment or concurrent opiate detoxification. The next largest group of patients were those admitted for conversion from methadone to buprenorphine. Fewer patients (*n* = 7, 4%) were admitted for benzodiazepine detoxification, detoxification from NPS or respite. Of the 31 patients with a diagnosis of benzodiazepine dependence, 20 (64.5%) also had a diagnosis of alcohol dependence; 26 patients (14.9%) had diagnoses of both alcohol and opioid dependence. Polysubstance misuse, here defined as being dependent on three or more substances, was present in 14 (8%) patients. There was no statistically significant association between either alcohol dependence or opioid dependence and chronic pain.

Two-thirds of patients (*n* = 107, 61.1%) had a psychiatric diagnosis in addition to their substance misuse diagnosis. The range of psychiatric diagnoses encountered is summarised in Table 2. Of these, the most frequent diagnosis was depression, present in 83 patients. Cognitive impairment was recorded for 10 (5.7%) patients, all of whom had alcohol dependence. A diagnosis of personality disorder was made in 11 (6.3%) patients. There was some comorbidity of psychiatric disorders, with 12 (6.9%) patients having two or more recorded.

**Table 2:** Psychiatric diagnoses (excluding mental and behavioural disorders due to psychoactive substance use)

Diagnosis	*n* (%)
Depression	83 (47.4)

Cognitive impairment	10 (5.7)

Emotionally unstable personality disorder	9 (5.1)

Anxiety disorder	8 (4.6)

History of psychotic symptoms	4 (2.3)

Post-traumatic stress disorder	3 (1.7)

Bipolar affective disorder	2 (1.1)

Schizophrenia	2 (1.1)

Mixed personality disorder	2 (1.1)

Postnatal depression	1 (<1)

Schizoaffective disorder	1 (<1)

Eating disorder	1 (<1)

Relatively coarse diagnostic categories have been used because of the nature of the patient population and the background information available to clinicians at the time of admission. For example, a number of patients reported or had a recorded history of psychotic symptoms. It is difficult to retrospectively attribute these reports to substance misuse, situational distress or other aetiologies. Similarly, although admission documentation identified a variety of anxiety disorders, these have not been sub-categorised because of a lack of discriminating detail.

**Fig. 1:**
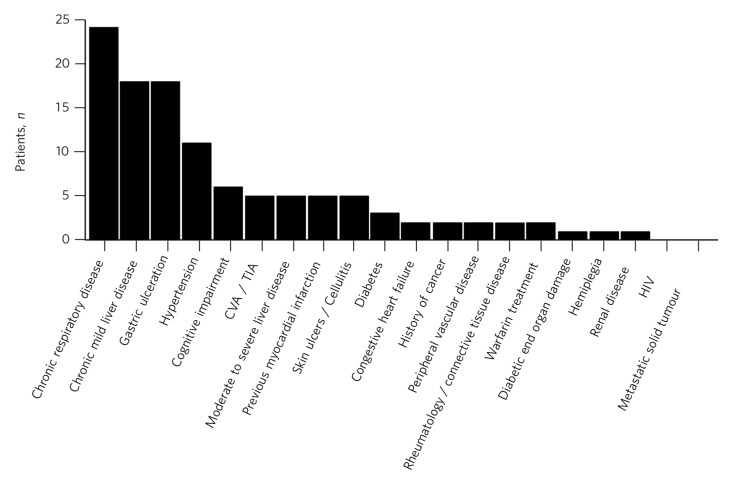
Comorbid diagnoses recorded in the study population CVA, cerebrovascular accident; TIA, transient ischaemic attack.

The proportion of patients with each of the CCI items is summarised in Fig. 1. The most common medical condition recorded was chronic respiratory disease, present in 46 (26.3%) patients, followed by 36 (20.6%) patients with gastric ulceration and 34 (19.4%) with chronic mild liver disease. Cardiovascular illnesses were also represented in the sample, with 21 (12%) patients who had hypertension, 9 (5.1%) patients who had experienced a previous myocardial infarction and 8 (4.6%) who experienced either a cerebrovascular accident or transient ischaemic attack. There were no patients in the sample diagnosed with HIV. Although we have categorised cognitive impairment as a psychiatric diagnosis in the present study, it is also included as a weighted item in the CCI, contributing to overall mortality.

**Fig. 2:**
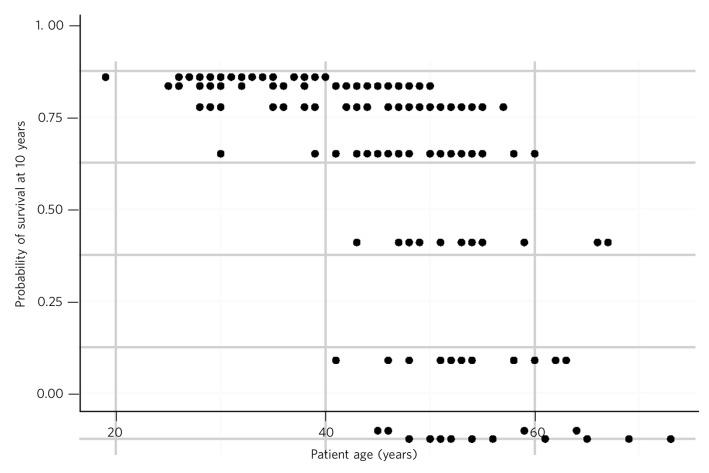
Predicted 10-year survival

Table 3 presents the distribution of CCI scores and associated 10-year mortality. The majority of patients had a predicted 10-year survival chance of greater than 75%. However, of the 29 (16.6%) patients with a less than 50% chance of survival at 10 years, a large number (55.2%) were aged 55 or less, and two were aged 45 or less. One patient aged between 36 and 45 had a predicted survival chance at 10 years of less than 20%. The breakdown of ages compared with predicted 10-year survival chance is illustrated in Fig. 2. There were 41 patients (23.4%) with a CCI of 4 or more, of whom 31 (17.7%) gained either two or fewer points due to age, thus suggesting multiple sources of physical comorbidity in these patients.

**Table 3:** Charlson Comorbidity Index (CCI) scores and predicted 10-year survival

CCI score	Predicted 10-year survival	*n* (%)
>5	<20%	16 (9.1)

5	20–49%	13 (7.4)

4	50–74%	12 (6.9)

3	75–90%	24 (13.7)

<3	>90%	110 (62.9)

## Discussion

The in-patient treatment unit is based in a large psychiatric hospital that is currently being redeveloped, and there has been considerable discussion regarding the future location of the unit, including the level of medical care required. Our research objectives emerged from informal discussions among medical and nursing staff, reflecting on an anecdotal impression that the in-patient unit was treating patients with ever-increasing medical complexity. It is not uncommon for patients to be transferred directly to a general hospital at the point of admission, or shortly afterwards, owing to the severity of their physical condition. The data presented here support this impression.

The unit has based its admission criteria on a since-withdrawn guideline from the Scottish Intercollegiate Guidelines Network (SIGN) – guideline no. 74.^9^ It outlines the patient characteristics that are likely to make community detoxification suitable and, conversely, situations where in-patient detoxification would be prudent. Acute physical or psychiatric illness, undernourishment and the presence of confusion or hallucinations all feature in the list of characteristics that indicate in-patient detoxification. These selection criteria can be expected to skew the medical comorbidity of the population being studied. Quantifying this degree of comorbidity remains valuable for the planning of care, interventions and service structure.

Despite the majority of the sample having a predicted 10-year survival chance of greater than 75%, a sizeable minority are carrying a high burden of medical comorbidity. The number of patients of younger age present in this group is of particular concern. Our data show that for patients aged between 45 and 55, the mean predicted 10-year survival chance is 68.6%. By way of comparison, the predicted 10-year survival chance for a 45-year-old Scottish man, based on 2011–2013 data published by the Office for National Statistics, is 96.6%. This falls to 91.9% at age 55 and to 80.6% at age 65.^10^

Previous work has shown the extent to which mental illness shortens life expectancy. A study conducted in a London sample showed that having a psychiatric diagnosis resulted in 8.0 to 14.6 life years being lost for men and 9.8 to 17.5 life years being lost for women.^11^ That study included people with ‘serious mental illness’ (schizophrenia, schizoaffective disorder and bipolar disorder) as well as those with a substance use disorder and depressive episode/disorder. The predictive tool used in our study does not allow for direct comparison, but it does highlight a similar impact on mortality.

Harmful use of alcohol is classically associated with gastrointestinal disease, cardiovascular disease and neurological damage. However, the most common physical illness recorded in this patient group was chronic respiratory disease. Most (78.9%) of the sample were current smokers at the time of admission. Smoking status was not included in the initial data collection tool and was recorded from retrospective review of electronic patient records and paper case notes where needed; we were unable to determine smoking status for one patient. In our sample, 49 (35.5%) of those who smoked were referred to the hospital smoking cessation nurse for assessment and possible intervention. There is some evidence that providing concurrent smoking cessation treatment during both out- and in-patient alcohol detoxification may be more beneficial than delaying it.^12,13^ With the recent smoking ban on the grounds of Scottish hospitals,^14^ this may merit more robust intervention in this population.

There is an established association between opioid dependence and infection with blood-borne viruses.^15^ Despite the significant number of patients with opioid dependence, the data presented here showed no patients who were diagnosed with HIV, which may reflect the success of opioid-replacement programmes. Of the 44 patients with liver disease, 10 (22.7%) had a diagnosis of opioid dependence, but only one of these did not have comorbid alcohol dependence. Screening for blood-borne viruses is, however, routinely offered to all patients admitted, given that hepatitis C remains an important and potentially treatable cause of cirrhosis and liver cancer, along with alcohol and metabolic syndrome due to obesity.^16^

The high number of patients seen here with chronic pain is consistent with previously published research.^17^ There is evidence that physical pain may predict lapse to heavy drinking, both before and after treatment,^18^ which may merit further investigation in this high-risk population.

Common to all systems of comorbidity scoring, the CCI provides a single number that is useful for patient-to-patient or group comparisons. In this case, the number is used to calculate a probability of survival at 10 years. Although this may be useful in demonstrating the stark nature of health outcomes in the present study population, it does not provide a sensitive measure of predicted quality of life or functional impairment.

### Limitations

This study was conducted within a single in-patient service and did not seek to modify routine clinical practice. Although based on a small sample size, the results are likely to be generalisable to similar in-patient units that use the criteria set out in SIGN guideline no. 74 to select the population for treatment.

Five doctors completed data collection forms, but the majority were completed by a study author (D.M.) who was employed as a psychiatric trainee on the unit for 4 months of the study period. There is the possibility here of observer bias, although all patients were discussed daily at the ward meeting with the consultant psychiatrist responsible for the unit. The same consultant verified substance misuse and psychiatric diagnoses. The population data presented show association rather than cause and effect. Other factors that have an impact on life expectancy, for example social deprivation or smoking status, were not recorded during data collection or controlled for in the data analysis.

### Implications

We showed that individuals receiving in-patient treatment for substance misuse carry a high burden of physical illness. This has implications for how services are delivered. While a general medical setting is equipped to provide limited, usually opportunistic, detoxification for patients with a high burden of physical comorbidity, it is not usually able to offer planned, evidence-based treatment for patients with a range of substance misuse disorders. In planning and providing for substance misuse treatment services, our data suggest that in-patient units with active medical input, resident medical cover and effective liaison with general medical hospitals continue to address a significant need. The ability to offer safe and effective treatment relies on ongoing training and adequate medical resources and equipment. The in-patient unit presented here provides physical health awareness groups on a weekly basis, but our data suggest that a more intensive approach may be appropriate. In particular, the very high rates of smoking and respiratory illness represent an area of potential intervention beyond that already offered within the hospital.

Given the significant overlap of physical and substance misuse diagnoses, we support ongoing efforts to raise awareness among psychiatrists of their patients' physical health needs. Similarly, we argue for ongoing efforts to provide substance misuse assessment and treatment skills to physicians.
